# A review of recent advances in plant-pathogen detection systems

**DOI:** 10.1016/j.heliyon.2022.e11855

**Published:** 2022-11-28

**Authors:** Rhea Patel, Bappa Mitra, Madhuri Vinchurkar, Andrea Adami, Rajul Patkar, Flavio Giacomozzi, Leandro Lorenzelli, Maryam Shojaei Baghini

**Affiliations:** a Center for Research in Nanotechnology and Science, Indian Institute of Technology Bombay, Mumbai, 400076, India; b Center for Sensors & Devices, Fondazione Bruno Kessler (FBK), Trento, 38123, Italy; c Department of Electrical Engineering, Indian Institute of Technology Bombay, Mumbai, 400076, India

**Keywords:** Agriculture, Pathogen detection, Miniaturized systems, Biosensors

## Abstract

Worldwide, a substantial economic loss in agricultural products is caused by plant pathogens. The increased losses in agriculture have drawn attention towards the development of miniaturized pathogen detection systems for phytopathology. This review paper's main selling point supports recent research (from 2015 to 2022) and technological advancements in the field of plant pathogen detection. The article discusses in depth important developments in the loop-mediated isothermal amplification (LAMP) assay, microfluidics, Molecular Imprinted Polymer (MIP) based biosensors, digital droplet PCR (ddPCR), disposable all-printed electronics, and nanoparticle-based sensors for instantaneous pathogen detection in agricultural applications. Utilizing nanoparticles to identify agricultural pathogens is a crucial topic that is explored. A brief on various commercially available detection systems worldwide have been listed. Finally, we discuss the perspective in the development of portable miniaturized systems and novel assay technologies based on advanced nanomaterials. Gold standard techniques: Although Polymerase Chain Reaction (PCR) and culture counting have been widely used for plant pathogen detection, they are not appropriate for measurements made in the field due to their higher installation costs, lack of portability, need for well-equipped laboratories, and requirement of skilled personnel. Therefore, these recent trends are overtaking the traditional methods in Agri-diagnostics because of their superior performances and suitability for the task.

## Highlights

• It's critical to identify plant diseases early to avert financial losses.
• Traditional methods have limitations.
• Recent advances in plant pathogen detection system.
• Application of nanotechnology in biosensing system.
• Commercially available devices for plant disease detection.

## 1 Introduction

Over the past 300 years, agricultural ecosystems have expanded to encompass around 40% of the planet's surface in order to meet the demands of every expanding population (Foley et al., 2011). The deterioration of plant products, which is estimated to cause a loss of between 10 and 30 percent overall, is one of the main constraints on food resources worldwide (Okawa, 2015). Since one of the major reasons for this deterioration is pathogens and their associated toxins, food safety is an issue to be considered. Bacterial, fungal, and viral infections are major causes of plant diseases. These infections spread widely across plantations by the introduction of the disease-causing agents into the field or through the infected plants (Sankaran et al., 2010). In basic and applied plant research, it's critical to make an early assessment of plant disease incidence and severity on field crops (Gautam & Kumar, 2020). It is necessary to conduct accurate and timely assessments since they serve as the foundation for field plant protection actions.

Different sensing methods have been applied throughout the years to develop sensitive and selective detection systems, from the most basic detection of symptoms emerging on leaves to the nucleic acid detection approaches. The direct and indirect methods of detection have been classified as the two main categories of conventional analytical techniques for the detection of plant diseases. Polymerase Chain Reaction is one of the direct techniques (PCR) (Fraaije et al., 2017), immuno-assays (Hewison and Kuras, 2005), and culture colony counting (Gautam and Kumar, 2020). The indirect methods are non-invasive techniques which include thermography, gas chromatography, hyperspectral imaging and fluorescence imaging (Fang and Ramasamy, 2015). These standard techniques are time-consuming, expensive, and labor-intensive (Khater et al., 2017). While direct methods show results with high sensitivity and high throughput analysis, such techniques are limited inside laboratories. The process involves tedious sample preparations, and skilled personnel to carry out measurements and analysis. The employment of an expensive camera system and extensive data analysis has made indirect methods beneficial for the early detection of plant infections, but this has limited their applicability to monitoring in larger canopies. In this paper, various sensors recently developed for plant pathogen detection have been reviewed. In this study, a systematic search was carried out by using the keywords – “plant diseases, biosensors, advances in agriculture, miniaturised devices for plant pathogens” in databases, including PubMed, Web of Sciences, and Scopus. We provide insights into various techniques, plant pathogens being detected, and the various transduction mechanisms of the biosensing systems. The recent methodologies developed along with the target details, and limits of detection are tabulated in Table 1 below. This paper takes a close look at recent technological advancements that are being utilised, or have the potential to be applied, to in-field direct diagnosis, which can simplify how farmers and pathologists diagnose plant diseases both now and in the future. Finally, the challenges on plant sensors as well as the future prospects are discussed.

**Table 1:** Performance comparison of different plant pathogen detection techniques.

Method of Detection	Detected Pathogen	Minimum detectable quantity	Sample type and host	Time required	Reference
Droplet Digital PCR (ddpcr)	*Botrytis cinerea*	2.67 copies/μL of DNA	Field samples	≈min	Suarez et al. (2005)
DNA amplicon detection using quartz crystal microbalance	*Botrytis cinerea, Pseudomonas syringae, And Fusarium oxysporum*	10^3^–10^4^ CFU	Lab samples	40 min	Khater et al. (2019)
Multiplex detection Method using RPA and SERS	*Botrytis cinerea, Pseudomonas syringae, a*nd *Fusarium oxysporum*	2 genomic copies of pathogen DNA	Field samples	40 min	Lau et al. (2017)
Loop-mediated isothermal amplification (LAMP)	*Pseudomo nae syringae pv.*	1.61 × 10 fg/μL	Lab and field samples	60 min	Chen et al. (2020)
	*Penicillium oxalicum*	100 pg genomic DNA	artificially/naturally contaminated	60 min	Vogt et al. (2017)
	*Phytophthora agathidicida*	1 fg DNA	Field samples	60 min	Winkworth et al. (2020)
	*Neofabraea perennans*	0.001 ng/μL	Artificially inoculated apples	10 min	Enicks et al. (2020)
	*Sclerotinia sclerotiorum*	10 pg of DNA	Artificially inoculated soil samples	Less than 60 min	Grabicoski et al. (2020)
Reverse transcription loop-mediated isothermal amplification (RT-LAMP)	Prunus necrotic ringspot virus (PNRSV)	10^−6^ of cDNA	RNA extracted from tomato Torrado virus-infected plants	–	Zong et al. (2014)
Molecular Imprinted Polymer (MIP)	*Neisseria meningitidis*	9.87 ng/mL	B Protein of *N. meningitidis*	–	Gupta et al. (2016)
Quantum dot-(QD) based biosensor	Citrus tristeza virus (CTV)	220 ng/mL	Citrus fields.	–	Shojaei et al. (2016)
Lateral Flow Immuno Assay	Potato virus Y	330 ng/mL to 5.4 ng/mL	Field samples	15 min	Razo et al. (2018)
	Stone fruit and almond infective bacteria	10^4^ CFU/mL	naturally infected plants	15 min	López-Soriano et al. (2017)
AuNP-based Local Surface Plasmon Resonance (LSPR) colorimetric nano-sensors	Tomato yellow leave curl virus	5 ng of infected plant's DNA	TYLCV diseased plants	–	Razmi et al. (2019)
Impedimetric biosensor based on gold nanoparticle	*Citrus Tristeza Virus* (CTV)	0.1 μM–10 μM	–	–	Khater et al. (2019)
Primer-mediated asymmetric PCR with AuNPs	*Pythophthora infestans*	0.1 pg/μM	Phytophthora *infestans* samples	1.5 h	Zhan et al. (2018)
Multiplexed imaging LSPR assay	*Fusarium* toxins	15 mg/kg	Lab samples	17.5 min	Hossain and Maragos (2018)
Label-free amperometric immune-sensor	Capsicum chlorosis virus (CaCV)	800 to 1000 times more sensitive	Field samples	50 min	Sharma et al. (2017)
Optical biosensor based on atomic layer deposition of ZnO	Grapevine virus A-type (GVA) protein	1 pg/mL to 10 ng/mL	–	–	Tereshchenko et al. (2016)
Magnetic immunoassay	Grapevine fanleaf virus (GFLV)	6 ng/mL to 20 μg/mL	Artificially inoculated plant samples	less than 30 min	Rettcher et al. (2015)
Amperometric	*Botrytis cinerea*	3 mg/L – gluconic acid 35 mg/L- glycerol	*Botrytis cinerea* isolated from naturally infected table grapes	0.5–2 min	Cinquanta et al. (2015)

## 2 Modern biosensor technology for plant pathogen detection

### 2.1 Loop-mediated isothermal amplification (LAMP) assay

**Figure 1:**
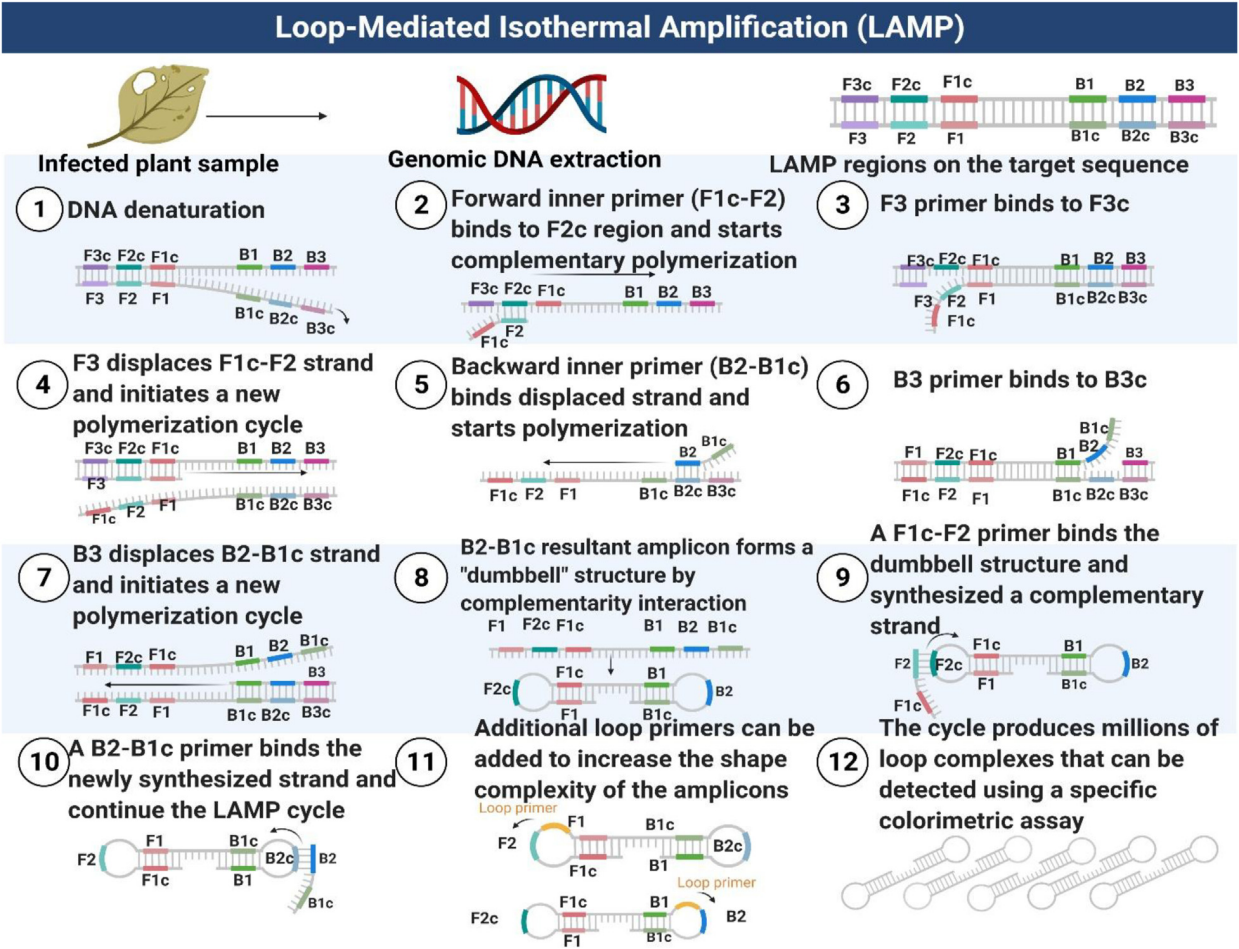
Principle of LAMP assay.

Traditional method such as PCR involves processes requiring DNA extraction from the tissue of a plant, and thermal cycling of the enzyme *Thermus aquaticus* polymerase to detect and quantify the pathogens affecting the plant. This is a major drawback limiting application of this process outside the laboratory. LAMP is a single-tube reaction method that uses an isothermal polymerase to amplify DNA at a particular temperature. When doing a LAMP test, a group of four to six different primers are used, and they bind to six to eight distinct primers on the target gene. The primer set comprises of loop forward and loop backward primers, as well as the two outer primers, F3 and B3, the Forward Inner Primer (FIP), and the Backward Inner Primer (BIP). The Bst DNA polymerase enzyme is used in the reaction, which occurs under isothermal conditions (Wong et al., 2018). The illustration of the principle of LAMP assay is given in Figure 1.

LAMP assays have been exploited as a recent advancement in plant pathogen detection. A LAMP test has been created to identify the tomato and potato pathogen *Phytophthora infestans*, which causes late blight on potatoes (Ristaino et al., 2020). The created assay was contrasted with traditional PCR, real-time LAMP, and ddPCR. The SYBR Green real-time LAMP assays were discovered to be 10 times more sensitive than traditional PCR and were specific for *P. infestans* in potato and tomato. For the purpose of finding *Pseudomonas syringae PV*, a team created the LAMP technique. The tomato (Pst), which causes tomato bacterial speck (Chen et al., 2020). Two backward loop primers, two internal primers, and two external primers (F3/B3) were all incorporated in the design of the hrpZ gene (B-Loop). The LAMP detection of Pst was optimised for a 60 min completion time at 63 °C. The Limit of detection (LOD) for bacterial suspension without DNA extraction was 1.05 × 10^3^ CFU/mL and for genomic DNA was 1.61 × 10 fg/L. A LAMP assay for *Penicillium oxalicum*, for fungus detection on grapes that have been intentionally infected, was able to provide a LOD was 100 pg genomic DNA per reaction. LAMP assay for the detection of *Phytophthora agathidicida-a* root rot causing oomycete for kauri dieback was developed most recently (Winkworth et al., 2020). The entire *P. agathidicida* DNA LOD was determined to be 1 fg. Similarly, a LAMP-based system developed for detection, which causes Bull's eye rot (BER) with a reaction time of 10 min, one can detect DNA quantities as low as 0.001 ng/L (Enicks et al., 2020). The detection of *Sclerotinia sclerotiorum*, a widespread plant pathogen with a LOD of 10 pg of DNA, has been reported using a quick, sensitive, and focused LAMP-based test (Grabicoski et al., 2020). Highly specific LAMP primers for *Fusarium proliferatum* are appropriate for the TEF-1 region (Wang et al., 2020). The same group also described the LAMP assay for quick detection of *F. proliferatum* for the diagnosis of ear and kernel rot in maize. *Colletotrichum nymphaeae* strain CCTUCch32 infection in asymptomatic strawberry plants was easily identified by a LOD of 50 fg/L DNA isolated from infected leaves (Karimi et al., 2019).

Most recently LAMP assays have also seen their application in the detection of RNA by using the enzyme reverse transcriptase which synthesizes complementary DNA from RNA, followed by its amplification using a polymerase. In another study, by creating a set of four primers based on the conserved sequences of the coat protein for identification of Apple chlorotic leaf spot virus (ACLSV) was detected using reverse transcriptase LAMP test. It was successfully used for on-site diagnostics with the assay's LOD of 0.02 g/L at 64 °C (Peng et al., 2017). A group described a highly specific, single-step RT-LAMP technique for quickly detecting 35 isolates of Indian citrus ringspot virus (ICRSV). To assess the virus's target of the coat protein gene, they utilized four different primers (Kokane et al., 2021). The detection was based on visualization of change of color after adding SYBR Green I during the process (Tseng et al., 2021), developed a test to identify six different viroids using one-step RT-PCR and RT-LAMP with ubiquitous primers – columnea latent viroid (CLVd), pepper chat fruit viroid (PCFVd), potato spindle tuber viroid (PSTVd), tomato apical stunt viroid (TASVd), tomato chlorotic dwarf viroid (TCDVd), and tomato planta macho viroid (TPMVd). The outcomes showed that six pospiviroids from solanaceae seeds and plants could be amplified using a pair of degenerate primers in a one-step RT-PCR. Depending upon the virus of interest, only 1 fg to 10 ng of viroid RNA can be used for detecting the virus successfully. In addition to these, LAMP assays were developed to detect needle blights which are fungal pathogens affecting the natural pines. For this assay, species-specific fluorescent probes for the three main infections that were of concern were created: *Lecanosticta acicola*, *Dothistroma pini*, and *Dothistroma septosporum*. The LOD of the assay was 0.128 pg/μL for *L. acicola*, 0.64 pg/μL for *D. pini*, and 3.2 pg/μL for *D. septosporum* (Agietti et al., 2021). A quick LAMP assay was proposed to detect *Marssonina coronaria* which causes apple blotch in most apple fields was recently developed (Ren et al., 2021). The primer designed was a ribosomal DNA internal transcribed spacer which was distinct from other apple disease-causing fungi. The LAMP test had a detection time of 70 min and a detection limit of 100 fg/L of *M. coronaria* genomic DNA. A group pf researchers recently developed a rapid virus diagnostic method based on LAMP assay using six primers to detect Sri Lankan cassava mosaic virus (SLCMV). The sensitivity of the developed assay compared to the standard PCR, was up to 10,000 times greater and could enable in early diagnostics of this plant disease. Also, using chemicals from a commercial kit that were intended to be stable when dried, a technique was created that enabled for on-the-go analysis without the use of a thermal cycler (Uke et al., 2022). A reverse transcription LAMP test for identification of Wisteria vein mosaic virus which affects wisteria-a woody, deciduous, ornamental flowering plant was studied. This assay had 100 times higher sensitivity compared to traditional PCR. The coat protein gene sequence of the wisteria vein mosaic virus served as the basis for the development of the RT-LAMP primers (Yao et al., 2022). The genome of the prune dwarf virus's coat protein region was the focus of a single-step colorimetric RT-LAMP test. The investigation was successful in obtaining a naked eye observation of positive LAMP reactions, where the colour changing from pink to yellowish signals a positive result of the virus infection in prunes. This method's sensitivity was 10 times more than that of traditional PCR (Çelik, 2022). Another team of researchers developed a LAMP assay to identify the pea enation mosaic virus (PEMV), which infects plants belonging to Leguminosae, using specific primer sets as inner- and outer primers. Their results showed up to 10^−6^ cDNA (Kim et al., 2022). A chapter describes how to use the immunological capture RT LAMP test (IC-RT-LAMP) to pinpoint a particularly virulent strain of a plant virus in field samples. The development of an IC-RT-LAMP test included the addition of an IC step to lower diagnostic costs and boost throughput. The two most common viruses that infect lily plants worldwide, lily symptomless virus (LSV) and cucumber mosaic virus (CMV), were both detected using the assay. Without isolating RNA, antibodies (anti-rabbit IgG) against recombinant LSV or CMV coat proteins were used to capture the viral antigens, which were then detected by RT-LAMP. Compared to traditional PCR, our assay was 100 times more sensitive. The method used to make the approach visible to the unaided eye (Zhang et al., 2021).

### 2.2 Microfluidics

Microfluidics is a multidisciplinary approach to manipulation of fluids in a small volume. Microfluidic chips can be coupled with different techniques such as PCR, LAMP, electrochemistry, and serve as a platform for automation and diagnosis. The DNA of *Ganoderma boninense*, a pathogenic fungus that infects oil palm trees in Malaysia, was extracted using a microfluidic device. The confirmation of excitation peaks was done using UV–vis spectroscopy analysis. The peaks were observed at 260–280 nm. Peak at 260 nm indicated the synthetic ssDNA being a pure sample whereas shift in peak to 280 nm indicated impurities of sample with other molecules. Current-voltage measurement results showed the accuracy of microfluidic devices for the real and synthesized samples of *G. boninense*. UV–vis studies and current-voltage measurements demonstrated effective DNA extraction (Ayoib et al., 2020). With a pretreatment channel, inertial impactor, and low pressure collection chamber to enrich the airborne fungal spores, a microfluidic chip was created for the real-time monitoring of crop fungal disease. Experiments and numerical analysis were both done to find the best design for the microfluidic device. Collected bio-aerosols were stacked radially in the pre-treatment channel. The initial impactor served the purpose of separating the collected bio-aerosols based on their sizes. Last but not least, the low-pressure collection chamber made sure that the aerosols wouldn't bounce (Wang et al., 2020). *Botrytis cinerea* and *Didymella bryoniae*, two DNA targets for fungal diseases, were used in the development of a reusable microfluidic bioassay. Thermal denaturation of DNA was then performed to regenerate the oligonucleotide sequence for its reuse. A quick assessment of multiple target detection was done in less than 10 min using the laser-induced fluorescence detection technique (Qu et al., 2017). An enzymatic colorimetric test for the identification of diseases in grapes using azelaic acid as the target moiety was developed. Thin-film silicon photosensors for optical detection were integrated into the microfluidic platform. Tyrosinase was immobilised on the sensor and inhibited AzA by immobilising it. The limit of detection of 5–10 nM range with the assay time of 10 min was reported (Brás et al., 2019). A disposable microfluidic device based on magnetic beads decorated with horseradish peroxidase and antibodies was developed. Citrus tristeza virus was found in samples from healthy and infected plant samples using the immunosensor and immunomagnetic separator, with a LOD of 0.3 fg/mL (Freitas et al., 2019).

**Figure 2:**
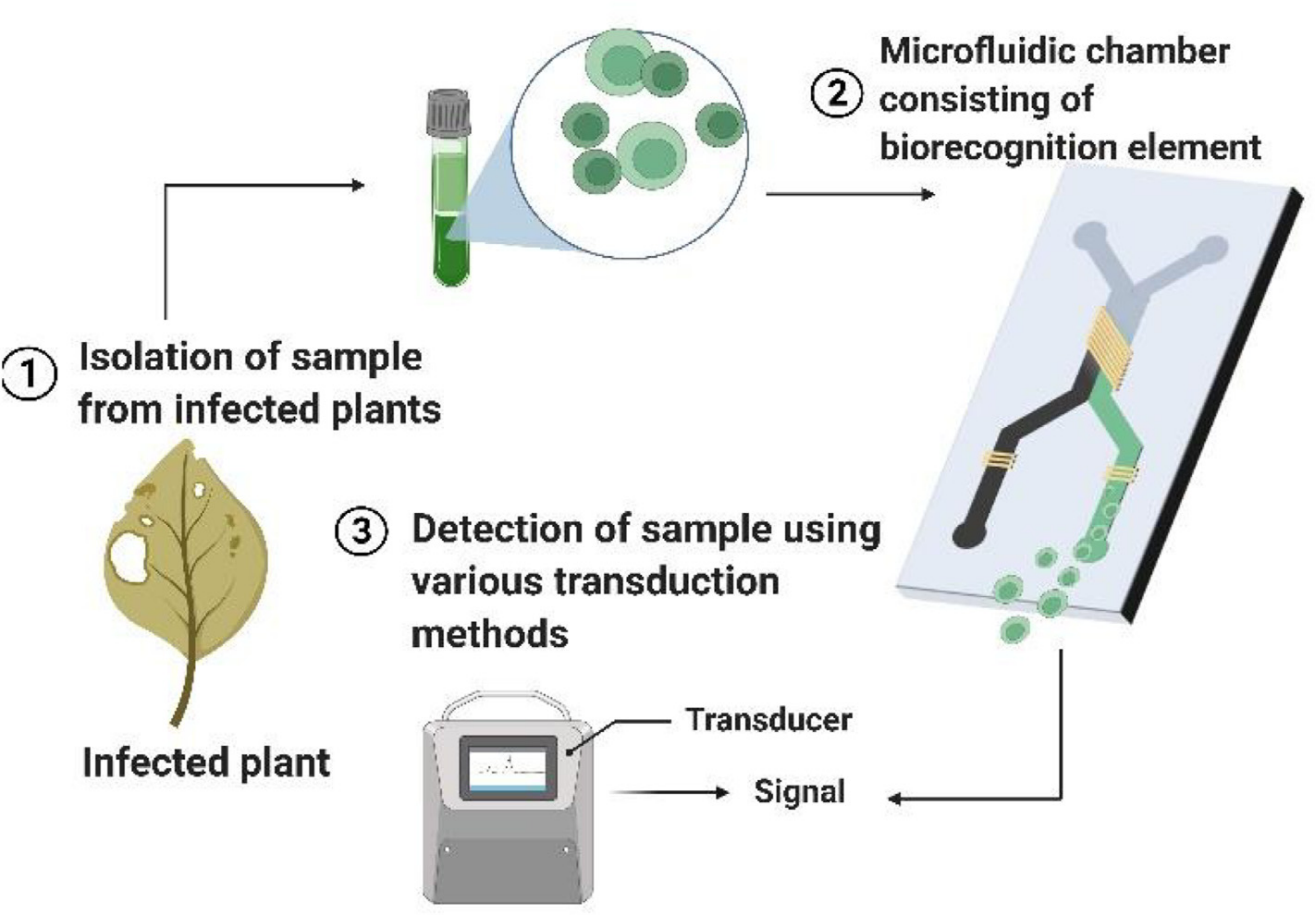
Diagram of a microfluidic device for identifying plant diseases.

A microfluidic device coupled with buried optical fibres to detect virus-infected *Phalaenopsis amabilis* leaves and flowers was another advancement. Polydimethylsiloxane (PDMS) was used to create a microfluidic stirring device, and optical fibers were inserted into it to improve the optical signal for detection. The device was capable of RNA isolation and purification. Amplification of nucleic acid was done using RT-LAMP. Infection of Capsicum chlorosis tospovirus (CaCV) on capsicum was tested as an example of the idea (Lin et al., 2015). A point-of-care portable microfluidic system that uses a thin-film amorphous silicon photodiode to assess transmission owing to optical transduction by adjusting the resistors in the circuit, the prototype also allowed the sensor to read out in both high and low light conditions. In a portable arrangement, the gadget was capable of measuring currents as low as 10^−13^ A. A peristaltic pump for moving fluids inside the channel as well as an LED light source might also be controlled by the system. To show the prototype's use in the portable detection of fungal diseases in plants, it was put to use in a bioassay (Bras et al., 2020). Figure 2 presents a basic schematic of a microfluidic system used for phytopathogen detection.

### 2.3 Molecular imprinted polymer-based biosensor

Molecular Imprinted Polymer (MIP) imitates the form and behaviour of a biorecognition element such that the receptor binds to it with high sensitivity and selectivity. A broad class called biomimetics deals with the study of chemical synthesis of such molecules which imitate natural molecules (Lin et al., 2018). The advantages offered by MIPs are their simple preparation, stability and low cost. In addition to that, they offer superior physical/chemical stability, greater shelf life, as well as reusability. There are numerous techniques to prepare MIPs. Chemical, photochemical, or electrochemical techniques can be used to eliminate target templates. The resulting polymeric materials have unique recognition sites that complement the target molecules' characteristics in terms of their size, shape, and functional groups. Because of their stability, production, and application, MIPs have a number of benefits over antibodies.

**Figure 3:**
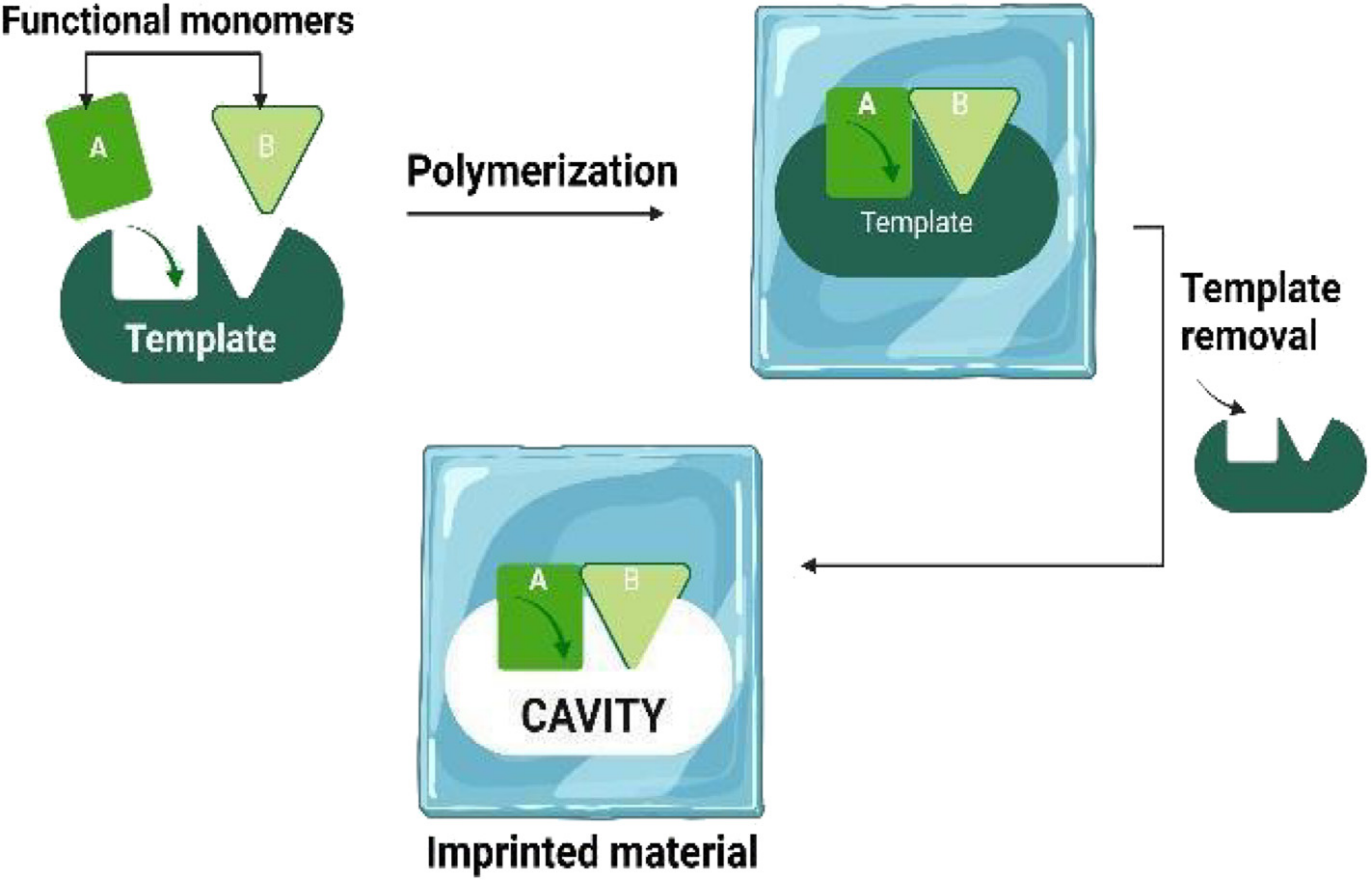
Diagram of the MIP preparation process.

For the purpose of selectively detecting a pathogenic bacterium utilizing electro-polymerization in one step, a bacteria-imprinted polypyrrole (BIP) film was created (Wu et al., 2018). This impedimetric technique involved microbiological template is removed after the BIP film has been applied to the glass carbon electrode (GCE). The deposition of MIP was done using electrochemical polymerization which allowed a controllable deposition of the polymeric film which offered a strong bond to the substrate. Conductive polymers of polypyrrole are frequently employed in the construction of electrochemical sensors due to its low nonspecific adsorption, excellent conductivity, exceptional stability, and easy polymerization under favorable circumstances. The created biosensor provided a broad linear range with great selectivity within 1.0 h for the detection of *Escherichia coli* O157:H7 for the development of electrochemical sensors is widely used because of its low nonspecific adsorption, good conductivity, superior stability, and efficient polymerization at mild conditions. The developed biosensor for *Escherichia coli* O 157:H7 detection offered a wide linear range with high selectivity within 1.0 h (Gupta et al., 2016). In another report, a detection of *Neisseria meningitidis* was detailed with the same procedure of electropolymerization to develop a film using 3-thiophene acetic acid. The film was developed on the electrochemical quartz crystal microbalance's gold-topped surface. An imprinting factor of 3.84 was achieved by the epitope-imprinted biosensor with a LOD of 9.87 ng/mL, demonstrating its viability for disease monitoring and early detection without preclinical therapies (Gupta et al., 2016). A schematic for MIP preparation is represented in Figure 3.

### 2.4 Lateral flow immunoassay (LFIA)

**Figure 4:**
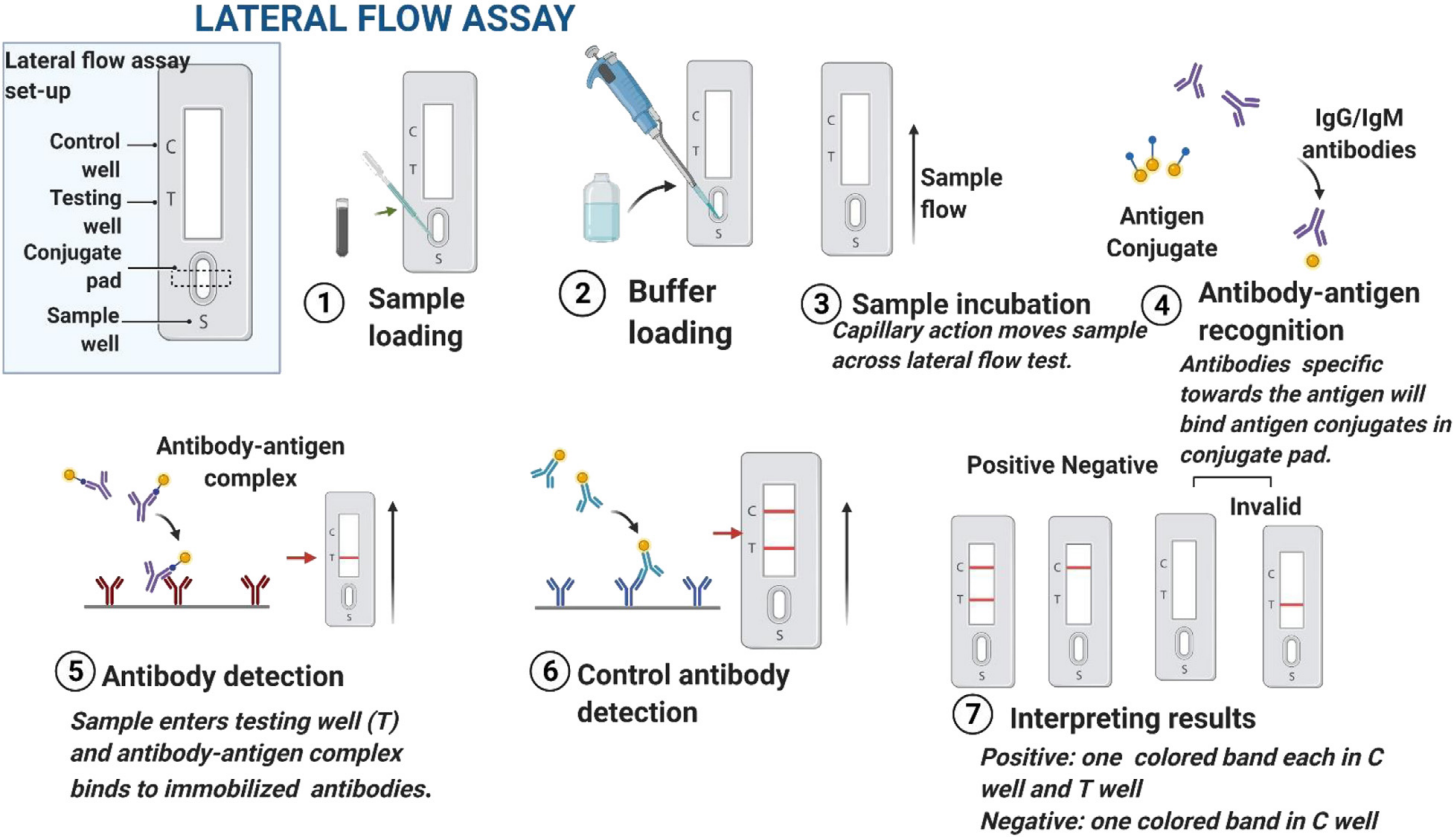
Process of a lateral flow assay.

The lateral flow assay (LFA), which uses paper as its foundation, is a platform for the detection and measurement of analytes in complicated mixtures. To achieve a very sensitive determination in LFIA, research was done on the production of a wide range of Au@Pt nanoparticles and their characterization for a peroxidase-mimicking activity. The study's target analyte was *Clavibacter michiganensis subsp. sepedonicus*, a common pathogen that causes tuber rotting in the field in potatoes and other crops. GNPs and Au@Pt nanoparticles were coupled with bacterial-specific antibodies. The LOD was 500 CFU/mL of potato tuber extract and 300 CFU/mL of buffer. Extraction, lateral flow, and colour enhancement (oxidation of diaminobenzidine by Au@Pt nanoparticles) were the three quick processes that made up the experiment. Without the need of specific tools or expertise, latent bacterial infections may be quickly detected using LFIA and the urchin Au@Pt nanoparticles (Panferov et al., 2020). In order to produce a polyclonal antiserum against BBrMV, a recombinant coat protein of the banana bract mosaic virus (BBrMV) was produced in *E. coli*. The expressed BBrMV coat protein's LOD was 10 ng, and the crude extract's detection limit was a 1:20 dilution with a reaction time of 5–10 min (Selvarajan et al., 2020). Similar to this, it was suggested to use repetitive cycles of DNA-RNA hybridization and the dissociation of fluorophores by Ribonuclease H to develop a sensitive fluorometric bio-barcode immunoassay for the detection of triazophos residue in agricultural goods and water samples. With a LOD of 0.0032 ng/mL, the competitive immunoassay displayed a broad linear range of 0.01–100 ng/mL. The signal amplification was carried by the ssDNA-AuNPs-mAbs. A pair of fluorophore/quenchers were added to the corresponding RNA probes at either end. RNA's phosphodiester bonds are hydrolyzed by the endoribonuclease RNase H (Wang et al., 2021). After the fluorophore and quencher on the RNA were separated, fluorescence emission was created. Surprisingly, this enzyme was unable to break down single-stranded or double-stranded DNA, only the RNA in DNA-RNA duplexes. As a result, the DNA-RNA hybridization is based on iterative cycles that enhanced the detection signal. An immunoassay to identify the pathogen *Tilletia indica* that causes the wheat-related illness Karnal bunt (KB). The covalent attachment of the *T. indica* antibody onto amine-functionalized diatom substrates, which served as the sensing platform, required the addition of glutaraldehyde. Diatom frustules made of a nanoporous, three-dimensional biogenic silica material demonstrated a special emission feature. The platform's usage of nanoporous 3-D biogenic silica frustules led to the observation of a bright, visible blue photoluminescence (PL) under ultraviolet (UV) stimulation. To discriminate between the *T. indica* (complementary) and *Aspergillus niger* (non-complementary) antigens, PL tests were carried out to determine the specificity and binding of the conjugated diatom platform (Mishra et al., 2020). The principle of LFIA has been shown in Figure 4.

### 2.5 Digital droplet PCR (ddPCR)

ddPCR is a sample partitioning technique that can identify just one copy of a gene. Its advantages over conventional PCR techniques are ease of use, reproducibility, and specificity in absolute measuring plant pathogens such fungi, bacteria, fastidious bacteria, viruses, and viroid (del Pilar Martínez-Diz et al., 2020). Based on PCR, an incredibly quick and accurate method able to detect up to 2 genomic copies of DNA of *Botrytis cinerea* has been developed (Suarez et al., 2005). Another example is the development of an isothermal amplification technology based on Recombinase Polymerase Amplification and lateral flow assay for the detection of *Candidatus Liberibacter asiaticus* that is sensitive, reliable, rapid, and inexpensive (Ghosh et al., 2018). In the creation of colorimetric nano-biosensing devices for pathogen detection, localised surface plasmon resonance using gold nanoparticles (AuNPs) have also been employed (Ghosh et al., 2018).

**Figure 5:**
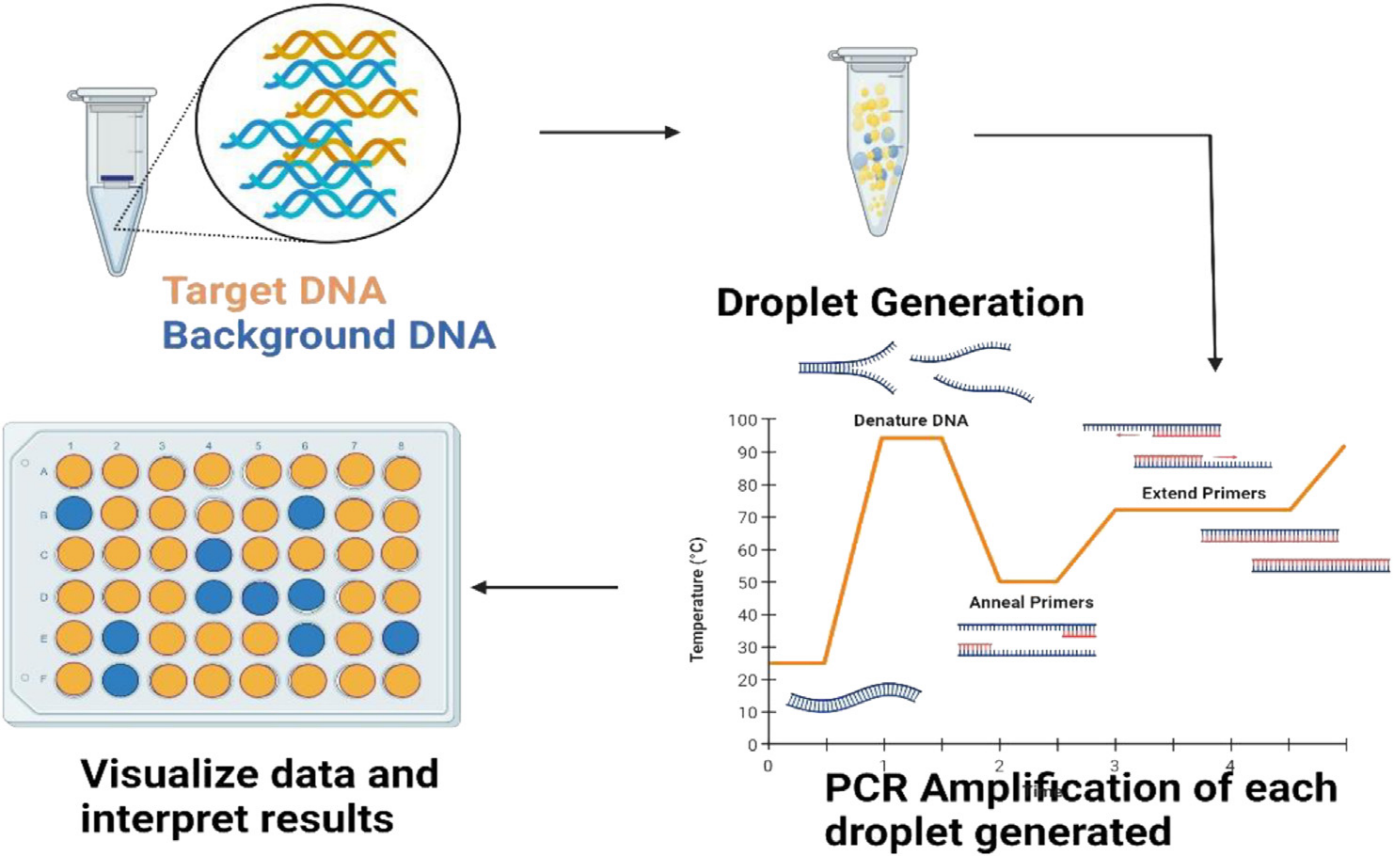
Working principle of ddPCR.

A modern method called droplet digital PCR (ddPCR) multiplies a single molecule in a droplet that is very diluted. To find the target molecule, a fluorescently tagged probe is employed. ddPCR owes to its low sensitivity to inhibitors and has proved as a consistent approach for even low target concentration detection. In this technique, the target sample is fractionated into 20,000 droplets, and each droplet's PCR amplification of the template molecules takes place. In comparison to PCR and real-time PCR, the ddPCR approach was claimed to be able to identify *Tilletia controversa teliospores* in soil for the first time with a sensitive LOD of 2.1 copies/μL (Liu et al., 2020). ddPCR method was also employed to identify *Ilyonectria liriodendra,* a causative agent of black foot disease afflicting with nurseries for grapevines (del Pilar Martínez-Diz et al., 2020). The authors claimed the technique was comparable with real-time PCR for its accuracy, efficiency, and specificity with the degree of correlation was calculated to be R^2^ = 0.95 with ddPCR. A research group developed a ddPCR format for a significant hazard to cucurbits, *Acidovorax citrulli*. The LOD was much more sensitive than qPCR of about 2 fg/DNA and 10^2^ CFU/mL bacterial cells (Lu et al., 2020). ddPCR against qPCR for quantifying An obligatory soilborne pathogen called *Plasmodiophora brassicae* causes clubroot infections in brassicca crops was developed. The proposed procedure was verified by calculating outcomes in various soil types. Results from their investigation demonstrated that ddPCR is a more flexible approach than qPCR for detecting and quantifying *P. brassicae* spores seeded (Lu et al., 2020). Another study, used qRT PCR and RT-ddPCR tests for identification of potato mop-top virus (PMTV) which is a threat to potato production worldwide. Both of the assays were able to detect PMTV in different soil types, however, RT-ddPCR showed a higher degree of linearity with RNA extracted from soil samples (Pandey et al., 2020). In another study, a viable ddPCR for the discovery of the fire blight-causing pathogen *Erwinia amylovora*, which affects apples and pears. The proposed technique included the chip-based QuantStudio 3D (QS3D). dPCR system for on field application (Santander et al., 2019). For the application, the internal transcribed spacer and the intergenic spacer from *Alternaria spp*. and *B. cinerea*, respectively, were employed. For each target, the detection limit was 0.1 pg of DNA. To comprehend pathogen detection at multiple plant growth seasons, the test was verified over a year (Larrabee et al., 2021). Figure 5 illustrates the working principle of ddPCR.

### 2.6 CRISPR based assays

The CRISPR/Cas system, also known as clustered regularly interspaced short palindromic repeat (CRISPR), is a group of CRISPR-associated proteins (such as Cas9, Cas12, and Cas13)., has been extensively used for plant pathogen detection via genome editing. In this technique, the target sequences are pre-amplified and CAS proteins are detected. Along with various isothermal nucleic acid amplification methods, CRIPR/CAS system can enable a system for point-of-care applications that can detect plant pathogens at low levels with excellent specificity and sensitivity (Zhang et al., 2022).

**Figure 6:**
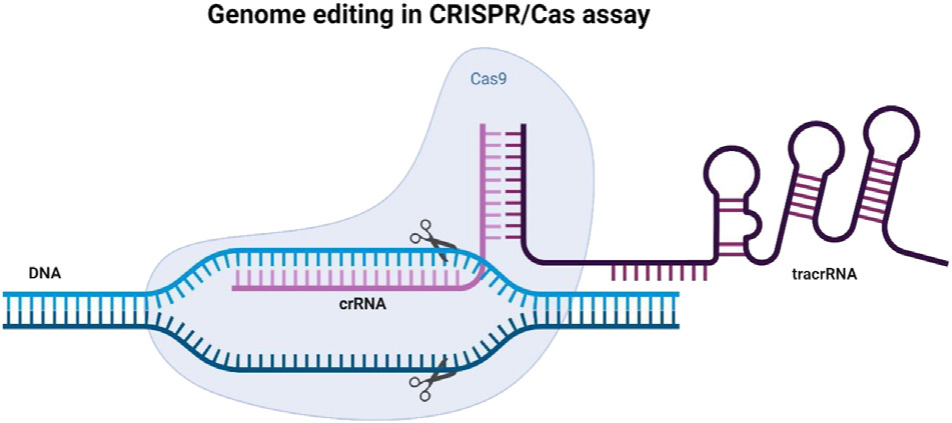
Illustration of CRISPR genome editing.

In one of the studies recently reported using CRISPR/Cas 9 system which triggered the isothermal amplification, they developed a visual assay to detect *Phytophthora infestans* genomic DNA. The AuNPs probes are further self-assembled and aggregated. Within a short period of time, the ensuing hue shift is evident. The ss RNA is very easy to use and has a straightforward design. This technique may be expanded to find other DNA sequences. While there is a linear relationship between the ratio of absorbance at 650 and 525 nm and the DNA concentration in the range of 0.2 pM–20 nM, the optical detection limit for DNA is 2 pM. The approach has substantially greater specificity with the single-base mismatch than the earlier CRISPR-based amplification platforms, and it can be visually readout (Chang et al., 2019). Figure 6. Illustrates the CRISPR process.

In order to identify plant RNA viruses, Aman et al. 2020 presented a quick RT-RPA approach with a CRISPR/Cas12a-based detection assay. Fusarium head blight (FHB) is a wheat yeild reducing disease caused by *Fusarium graminearum*. A CRISPR-Cas12a-based dual recognition technique was developed to detect this pathogen as low as 1 fg/μL of total DNA. A guide RNA hybridization and was screened to detect ITS region and transcription elongation factor (Mu et al., 2022). A team of scientists set out to find the Mungbean Yellow Mosaic India Virus, which affects leguminous crops, and the Ageratum Enation Virus, which affects weed crops, using Collateral Cleavage Independent CRISPR/Cas12a-based detection assay. The point-of-use diagnosis of tomato yellow leaf curl virus (TYLCV) and tomato leaf curl New Delhi virus was demonstrated by Mahas et al., in 2021 using a LAMP-coupled Cas12a technique. The diagnosis time was close to ∼1 h and the results could be interpreted using visualization from a basic, inexpensive fluorescence visualizer. In another study, CRISPR-Cas9 genome editing of *Venturia inaequalis*, a pathogenic agent of Apple scab. For the high-throughput screening of CRISPR-Cas9 gene-edited mutants of this pathogen, they presented HRM curve analysis (Rocafort et al., 2022).

The advantages and disadvantages of the techniques mentioned in this section have been summarized in Table 2.

**Table 2:** Benefits and drawbacks of various plant pathogen detection methods.

Technique	Benefits	Drawbacks
**LAMP Assays**	High efficiency, specificity, simplicity and quickness; Simple equipment requirement; Constant temperature and short reaction time.	Difficulty in primer designing; Complicated process for non-specialist.
**Microfluidics**	Small volume of sample requirement, portable, high selectivity and fast response	External parameters such as pH, temperature and pressure can affect the analysis
**MIP**	Low cost, quick preparation, reproducible and stable; Resistant to high pressure, high temperature, and extreme pH conditions; Long term storage possible	Needs reduction in non-specific binding; Large-scale production is difficult
**LFIA**	Fast, low cost, small sample volume; Point of care diagnostics; Longer shelf life and pretreatment is not often required.	Good antibody preparation is mandatory, analysis time relies on type of sample
**ddPCR**	High sensitivity, absolute quantification as it needs no extrapolation	Cost of the instrument for the initial purchase is high; Requires rigorous quality control plan
**CRISPR**	Simple design, high efficiency	Unexpected mutations due to wrong site cleavages

## 3 Nanotechnology in bio-sensing

The use of nanomaterials has resulted from developments in the field of nanotechnology and nanosensors for plant monitoring. Nanomaterial are structured to have a size of 1–100 nm in at least one dimension and show peculiar properties due to quantum confinement, and properties such as extremely low photobleaching, fluorescence in live tissue on low or clear backgrounds, and special magnetic/optical characteristics. Nanomaterials find its usage in optical biosensing and colorimetric detection schemes for these reasons (Guo et al., 2014). Nanomaterials have been exploited to enhance biosensors. The tailored size tuning of these nanomaterials in biosensors has resulted in high stability, selectivity, rapid dynamics, sensitivity, and reproducibility in plant pathogen detection (Giraldo et al., 2019). Nanomaterials have been employed to boost the analyte and surface contact in biosensors because of their higher surface-to-volume ratio (Kwak et al., 2017). In addition to that, the small size of nanomaterial allows these engineered nanomaterial to be embedded in plants for monitoring of signaling molecules which can be monitored in real-time. These substances can function as DNA scaffolds that can penetrate plant cell walls, opening the door for the creation of biosensors with genetically encoded information.

Biosensors are crucial for accurately and efficiently identifying molecules due to their high signal-to-noise ratio. However, one of the limitations is the instability and low signal strength for its efficient detection with sensitivity. Functional nanoparticles overcoming these limitations in conjugation with the bio-recognition element of the sensor could enhance the efficiency of the overall device. The highly sensitive biosensors coupled with nanoparticles are discussed through the overview of the recently reported research in this field. Khater et al. (2017) have previously summarized papers on the state and difficulties of several nano-biosensing devices for plant diseases. Based on the kind of nanomaterial employed in biosensors for plant disease diagnostics, this section is subdivided into subsections. The parts below have been covered.

### 3.1 Quantum dots-based biosensor

Quantum Dots (QDs) are fluorescent nano scale semiconductor crystals with a bright and tunable emission range. The emission range is set from visible region to the near-infrared region based on the size of quantum dots. Quantum dots offer a unique optical property known as fluorescence resonance energy transfer, which is the transfer of energy between two reactive molecules (Sanzari et al., 2019). This property is exploited by QT-FRET immunoassays for visual identification of pathogen infections. QD-FRET has been used to find phytopathogens like as *Aspergillus amstelodami, Candidatus Phytoplasma aurantifolia,* and *Polymyxa beta* (Safarpour et al., 2012).

Recent developments include an electrochemical ELISA for the detection of one of the most damaging pathogens of bananas- Banana bunchy top virus (BBTV). Cadmium selenide (CdSe) quantum dots (QDs) were used as signal amplifiers, which made the method appropriate for lab use. To standardize this assay BBTV and utilized antibodies produced in opposition to it. The diseased samples gave higher impedance in comparison to healthy samples due to the presence of pathogenic micro-organisms in the diseased sample (Majumder et al., 2020). A mini-review on the QD-based biosensors in plant virus detection can be found for further reading (Lee et al., 2018). The review describes the features of QD-based biosensors based on the physicochemical parameters of interface components and bio conjugates. Nanobiosensors for detection of *citrus tristeza* (CTV) based on the fluorescence emission of cadmium telluride quantum dots (CdTe-QDs) was developed in which CdTe-QD particles were conjugated to CTV coat protein (CTV-CP) corresponding antibody. Two specific and sensitive QD-based approaches-FRET biosensors and non-FRET-based biosensors for rapid detection of CTV infected plants were then discussed (Shojaei et al., 2016). Citrus tristeza virus (CTV) coat protein was immobilised on the surface of carbon nanoparticles (CNPs) using cadmium-telluride QDs coupled to an antibody. The CNPs muted the CdTe QDs' fluorescence after being immunobound by both the QD-Ab and the CP-loaded CNPs. LOD was determined to be around 220 ng/mL of CTV.

### 3.2 Metallic nanoparticle-based biosensors

Metallic nanoparticles are frequently utilised to create extremely sensitive electrochemical and optical biosensors because they improve electrical conductivity and optical signal. Surface plasmon resonance immunosensors for the detection of metals, such as AuNP, have been developed using metallic nanoparticles for detection of *T. indica* in wheat has been reported in the past (Singh et al., 2010). Similarly, copper nanoparticles have also been used to produce cheaper nanomaterials-based biosensors; overcoming the high cost of AuNPs synthesis. An electrochemical sensor was developed to monitor *Sclerotinia sclerotiorum* in oil seeds using modified gold electrodes with copper nanoparticles (Wang et al., 2010). Some recent work on biosensors using such nanomaterials has been reported below. An AuNP based immunochromatographic assay for detection of GLARaV-3, a pathogen causing a significant loss and decreasing quality of grapes has been discussed (Byzova et al., 2018). The influence of the size of the AuNPs is also discussed in the research, and it is demonstrated that decreasing the size of the gold nanoparticles improves the surface to volume ratio for analyte interaction, which in turn raises the sensitivity of the test. The immunochromatographic assay was prepared with AuNPs with 3 different diameters of nanoparticles. The obtained results showed the significance of the size of GNPs in the enhancement of the detection system as the GNPs with the smallest diameters showed 8 times more sensitivity to that of the largest GNPs used. The immunochromatographic assay also provided a rapid visual detection within 10 min. Similar to this, a quick method for lowering the lateral flow immunoassay's (LFIA) LOD for detecting potato virus Y in infected and uninfected potato leaves was suggested (Razo et al., 2018). The strategy comprised the binding of a GNP coupled with antibodies and a polyvalent antigen. When compared to standard LFIA, pre-incubating the GNP conjugates and samples for 30 s revealed a drop in the LOD from 330 ng/mL to 5.4 ng/mL. The development of an electrochemical biosensor for *Pseudomonas syringae* detection using disposable screen-printed carbon electrodes involved the use of AuNPs. The sensor was based on a recombinase polymerase reaction after a quick amplification of the target pathogen's DNA. Comparing the sensor's sensitivity to that of the traditional PCR gel electrophoresis technique, it demonstrated 10,000-fold greater sensitivity (Lau et al., 2017).

An unmodified AuNP-based LSPRc colorimetric nanosensors for the identification of TYLCV in infected plants was discussed by Razmi et al. (2019). The presence of target viruses could be done with visual inspection due to color change. The developed sensor could detect the TYLCV genome in 5 ng of infected plant's DNA with the need of amplification or detection instrument. An onsite nanosensor based on LFIA was designed for the identification of stone fruit and almond infective bacteria (López-Soriano et al., 2017). The pure cultures and spiked samples were having a LOD of about 10^4^ CFU/mL. The outcomes revealed a strong association between plate isolation and real-time PCR findings. Thus, the suggested immunosensor was proven to be an easy and affordable method for Prunus detection for on-site sample analysis. A group of researchers recently proposed an impedimetric biosensor based on AuNPs for detection of a nucleic acid of CTV. The biosensor was based on a SPCE whose sensing platform was modified by electrodepositing AuNPs to enhance the electrode conductivity. The sensor showed a logarithmic relation between the impedance values concerning CTV DNA concentration in the range of 0.1 μM–10 μM (Khater et al., 2019). *Phytophthora infestans* may be visually detected utilizing a new technique that makes use of lateral flow biosensors. AuNPs were combined with primer-mediated asymmetric PCR to create the biosensor. The biosensors could detect DNA concentrations as low as 0.1 pg/μM with high specificity within 1.5 h (Zhan et al., 2018). A multiplexed iSPR assay using AuNPs functionalized with secondary antibodies as a signal amplification tag was used in this work to detect three distinct Fusarium toxins (DON, T-2, and ZEN) in wheat. With a LOD of 15 mg/kg for DON, 12 mg/kg for T-2 toxin, and 24 mg/kg for ZEN, the antigen-coated biosensor platform shown good reusability (Hossain and Maragos, 2018). Lately, Sharma et al. (2017) developed a label-free immunosensor to detect Capsicum chlorosis virus (CaCV) in bell pepper leaves. The immunosensor was based on immobilizing virus antigens over AuNPs surface and multi-walled carbon nanotubes. The immunosensor was 800–1000 times more sensitive to CaCV detection using DAC-ELISA.

### 3.3 Biosensors based on metal oxide nanoparticle

CuO, ZnO, MnO, MgO, and TiO2 are examples of metal oxide nanoparticles that improve a sensing system's electrical, catalytic, and light absorption capabilities. Therefore, these metal oxide-based NPs is used in plant diagnostics by studying the interaction of metal oxide NPs inoculated in plants. Biosensors for identifying plant diseases have also been created using metal oxide NPs (Imada et al., 2015; Cai et al., 2018; Elmer et al., 2018; Liao et al., 2019). A novel optical biosensor based on atomic layer deposition of ZnO films for analysis of the grapevine virus A-type (GVA) protein was developed by Tereshchenko et al. (2016). The label-free biosensor's sensitivity to GVA-antigens was found to be between 1 pg/mL and 10 ng/mL. Similar to this*, Aspergillus niger* has been identified using CuO NPs (Etefagh et al., 2013).

### 3.4 Magnetic nanoparticle-based biosensors

Magnetic nanoparticles provide strong magnetic properties to the pathogens which otherwise cannot be found in those entities. Iron oxide magnetic nanoparticles which are coated with chitosan (CS-MNPs) were used to develop a colorimetric assay (Le et al., 2020). The surface CS-MNPS is positively charged which can interact with negatively charged surfaces of bacteria. This resulted in reduction of their peroxidase-like activity by hindering 2-2′-azino-bis (3-ethylbenzothiazoline-6-sulfonic acid) diammonium salt (ABTS) to the positively-charged CS-MNPs. This functions as a colorimetric test for finding bacterial cells. Grapevine fanleaf virus (GFLV) was created via immunofiltration, subsequent magnetic detection, and the quantification of magnetically tagged viral particles (Zong et al., 2014). Monoclonal GFLV capsid protein antibodies were used to immobilize infiltration columns and attach those antibodies to magnetic NPs. In less than 30 min, this study showed GFLV concentrations in the range of 6 ng/mL to 20 g/mL. With LOD of 2–60 ng/mL, the magnetic immunoassay was also used to identify Potato virus X and Tobacco mosaic virus.

A comparison between different recent methods concerning the method of detection and minimum detectable quantity employed in agricultural pathogen detection is summarized in Table 1.

## 4 Commercially available devices

The various commercially available devices/products for plant pathogen diagnosis are given in Table 3.

**Table 3:** Commercially available devices for plant pathogen detection.

Device/Product	Underlying principle	Company	Target application
AgriStrip	Lateral flow immunoassay	BIOREBA Switzerland	Pathogens of ornamental crops
PCR macro-array	PCR	BIOREBA Switzerland	Eight potato viral pathogens:
Rapid plant disease tests	Lateral fiow assay	Pocket Diagnostic, UK	*Erwinia amylovora*, Potato virus Y, Phytophthora, *Ralstonia solanacearum*
Alert Test Kits	Lateral fiow assay	Neogen Corporation	*Pythium, Phytophthora, Rhizoctonia*
ImmunoStrip	Lateral fiow immunochromatography	Agdia, Elkhart	*Plant virus*
Agdia products	ELISA, PCR,	Agdia, Elkhart	*Bacteria, fungus, oomycete, virus, viroids,*
SmartCycler	Real-Time PCR	Cepheid	*Aspergillus, Phakopsora* sp.
TwistAmp® Exo RT kit	Recombinase Polymerase Amplification (RPA)	-	*Yam mosaic virus* (YMMV)

## 5 Challenges and future outlook

Recently, sensor technology for pathogens detection has had a tremendous development to meet the challenge to detect and control plant/vegetable diseases by offering efficient technologies that can independently assess crucial biochemical variables. But because of a general mismatch in needs and a dearth of in-depth expertise that can address new and developing difficulties, there is a gap between the technology and the end-user (including the investigators and technicians that apply these technologies). The challenges of stability, sensor manufacturing cost, the time required for sensor response, etc are still not fully achieved. Contrary to the past, when the analysis of crops and their associated diseases was limited to plant pathologists, precision farming has provided simpler and farmer-friendly analysis in decision-making. Further, Artificial Intelligence (AI) and Machine Learning (ML) improved crop security by assisting decision-making processes and supplying a higher degree of automation. To broaden the monitoring to large areas with sufficient data coverage in space and time, the acquisition and gathering of good quality data from individual sensors need to be bridged to data analysis. The requirements on the sensing devices are particularly challenging when sensors have to be implemented in the wide-area monitoring systems such as Wireless Sensors Networks (WSN), in Unmanned Aerial Vehicles (UAV) for remote monitoring, and in IoT-based platforms for providing a new solution to enhance agricultural production. Moreover, due to the large amount of data collected, an easy-to-use and robust analytical model would be necessary to treat the data and provide a clear information to the end-user. For this reason, onsite data analysis is gaining attention and various data analytics techniques, including AI/ML, developed for other applications are now being applied to agriculture quality monitoring. Since the majority of conventional technologies are confined into laboratories and require experienced personnel and high time consumption, a growing trend in sensor miniaturization resulting in the development of biosensors can be seen. Lab-based techniques described in the direct methods are known to provide results with high sensitivity and selectivity. However, techniques such as PCR, LAMP, or RPA fail in distinguishing live cells from the dead ones, resulting in the wrong interpretation of results-a crucial parameter when the application of pesticides or insecticides in a sample is to be evaluated. Integration of AI/ML-based techniques for classification can lead to correct assessment in such cases. Biosensors have been used for on-field assessment of agricultural pathogens and have shown promising results and a lot of interest among researchers towards its further improvement. However, perturbance, biofouling, and passivation due to molecular metabolites, proteins, etc, and the chemical complex present in the sample matrix cause interferences with the biosensor performances. Therefore, sample pre-treatment, extraction, and filtration become necessary. The challenges and complexity of the mentioned biosensor requirements have to be evaluated when systems with a high level of automation are required. The use of POC testing devices has resulted in providing rapid results which have enabled farmers in taking quicker measures in taking decisions to minimize crop losses. Such methods have eliminated complex and multi-step sample preparation procedures, and have led to reducing logistic problems and transportation costs to the laboratories for assessment. However, the specificity of devices towards single pathogens and lower LOD is still a limitation. Future direction in such devices should be taken in a way such that multiple pathogens could be detected with lower limits of detection and reduced costs. Microfluidics based biosensors provide solutions to such requirements by allowing mixing, filtration, and detection on the same chip through manipulation of small volumes of liquid. The growing trend of research and progress in this field has shown its potential in terms of its real-time assessment and portability, and potential future works in terms of enhancement of multiple biosensors and multiple pathogen detection in a single chip platform. Usage of nanomaterials in biosensors has improved the sensitivity of biosensors but the introduction of such materials comes at a price of its high cost and its sustainability. Therefore, the search for cost-effective and environment-friendly materials for nanomaterial-based biosensors is undoubtedly a future opportunity that will lead to the commercialization of such biosensors.

## 6 Conclusion

This review sums up the recent usage of various sensing technologies in agricultural pathogen detection. The review highlights the advantages of miniaturized sensors in terms of ease of manufacturing, cost effectiveness, response time, and sensitivity thus making such sensors compatible for on-field measurements, and an obvious replacement for cumbersome lab-based measurement techniques. While in the case of on-field deployment of sensors, miniaturized biosensors provide an edge by providing higher throughput, sensitivity, and portability. Further improvement of such biosensors involves the usage of nanomaterials to enhance sensitivity and selectivity. The most recent trends in the field of microfluidics, digital droplet PCR, LAMP assays, lateral flow system, and the use of molecular imprint polymers have been discussed. Commercially available biosensors for agricultural pathogen detection have also been listed. The review paper concludes with a note on challenges existing in the current sensing technologies with future perspectives on possible enhancements to those techniques. Finally, a note has been penned upon the need for integrated smart sensor systems and networks on automated decision-making tools to allow either more control to the end-user or one potential possibility in this area is to give machine-based decision making.

## Declarations

### Author contribution statement

All authors listed have significantly contributed to the development and the writing of this article.

### Funding statement

This work was supported by INSPIRE (IF180964), Department of Science & Technology, India, India–Trento Program for Advanced Research (ITPAR – IV, INT/Italy/ITPAR-IV/MEMS/2018/G), Department of Science and Technology, India, European Union's Horizon 2020 research and innovation programme for the Marie Skłodowska-Curie grant agreement No 813680 (AQUASENSE).

### Data availability statement

Data included in article/supp. material/referenced in article.

### Declaration of interest's statement

The authors declare no conflict of interest.

### Additional information

No additional information is available for this paper.
